# PHB2 affects the virulence of Vip3Aa to Sf9 cells through internalization and mitochondrial stability

**DOI:** 10.1080/21505594.2022.2064596

**Published:** 2022-04-20

**Authors:** Baoju An, Yizhuo Zhang, Xuelian Li, Xiaoyue Hou, Bing Yan, Jun Cai

**Affiliations:** a Department of Microbiology, College of Life Sciences, Nankai University, Tianjin, China; b Jiangsu Institute of Marine Bioresources development, Lianyungang, China; c College of Food Science and Engineering, Jiangsu Ocean University, Lianyungang, China; d Key Laboratory of Molecular Microbiology and Technology, Ministry of Education, Tianjin, China; e Tianjin Key Laboratory of Microbial Functional Genomics, Tianjin, China

**Keywords:** Vip3Aa, PHB2, internalization, mitochondria

## Abstract

The vegetative insecticidal proteins (Vip3A) secreted by some *Bacillus thuringiensis* (Bt) strains during vegetative growth are regarded as a new generation of insecticidal toxins. Like insecticidal crystal proteins, they are also used in transgenic crops to control pests. However, their insecticidal mechanisms are far less defined than those of insecticidal crystal protein. Prohibitin 2 (PHB2) is a potential Vip3Aa binding receptor identified from the membrane of Sf9 cells in our previous work. In this paper, we demonstrated the interaction between Vip3Aa and PHB2 using pull-down, dot blotting, microscale thermophoresis, and co-immunoprecipitation assays. PHB2 is distributed on the cell membrane and in the cytoplasm, and the co-localization of PHB2 and Vip3Aa was observed in Sf9 cells using a confocal laser scanning microscope. Moreover, PHB2 could interact with scavenger receptor-C via its SPFH (stomatin, prohibitin, flotillin, and HflK/C) domain. Downregulation of *phb2* expression reduced the degree of internalization of Vip3Aa, exacerbated Vip3Aa-mediated mitochondrial damage, and increased Vip3Aa toxicity to Sf9 cells. This suggested that PHB2 performs two different functions: Acting as an interacting partner to facilitate the internalization of Vip3Aa into Sf9 cells and maintaining the stability of mitochondria. The latter has a more important influence on the virulence of Vip3Aa.

## Introduction

As the most widely used bio-insecticidal reagent, Bt could produce various toxins, such as crystal proteins (Cry) during sporulation [1], as well as vegetative insecticidal proteins (Vip) during the vegetative stage [2]. Cry toxins target midgut cells and form pores, resulting in cell osmotic lysis. They are used widely to control many pests [3]. However, cases of insect resistance to Cry toxins are constantly emerging [4–7]. As a strategy to delay the development of resistance, it is essential to find and apply insecticidal proteins that act via different mechanisms [8].

The Vip toxins are genetically and structurally distinct from known δ-endotoxins and have a broad insecticidal spectrum against Lepidopteran pests, making them as a new generation of insecticidal proteins [2,9]. The mode of action of Vip3A toxins is believed to be similar to that of Cry toxins. Vip3A is secreted from Bt in a protoxin form, undergoes proteolytic activation in the larva midgut, binds to receptors on the surface of midgut epithelium, and finally forms pores to trigger cell death [10–12].

Recent studies have proposed that the cell death caused by Vip3A might involve other mechanisms in addition to the pore formation model. Jiang et al. showed Vip3Aa induced apoptosis via a series of typical characteristics, including breakage of DNA, collapse of mitochondrial membrane potential, and activation of Sf-caspase-1 in Sf9 cells [13]. Hernández-Martínez et al. further confirmed that a sublethal concentration of Vip3 could upregulate caspase gene expression in *Spodoptera exigua* larvae and induce cell apoptosis [14]. Hou et al. indicated that Vip3Aa induces mitochondrial dysfunction and denaturation of lysosomes to promote Sf9 cell apoptosis [15].

In the process of Bt toxin action, the receptors play an essential role. Several proteins in midgut cells of Lepidopteran insects have been identified as receptors for Cry toxins, such as cadherin-like proteins (CAD), aminopeptidase N (APN), alkaline phosphatase (ALP), and ABC transporters [16,17]. In 2010, it was reported ribosomal protein S2 from a *Spodoptera frugiperda* (Sf21) cell line could interact with Vip3A toxin as chaperone protein but not as a receptor [18]. In 2011, active Vip3A was demonstrated to bind 55 kDa and 100 kDa unknown proteins in *Ephestia kuehniella* [19]. In 2018, two proteins, scavenger receptor-C (SR-C) and fibroblast growth factor receptor (FGFR) of *Spodoptera frugiperda* (Sf9) cells, were identified definitively as Vip3Aa receptors [20,21]. In 2019, a tenascin-like protein from *Agrotis ipsilon* epithelial tissue was isolated as a new receptor for Vip3Aa [22]. It is speculated that other Vip3A receptors remain to be discovered. In addition, compared with Cry toxins, there is still a lack of understanding of how Vip3A exerts toxicity through its receptors.

In our previous work, Jiang et al identified about 70 proteins from Sf9 cell membrane as potential receptors of Vip3Aa [20]. Among them, SR-C and FGFR were already verified as receptors for Vip3Aa. Here, we focused on another protein, PHB2, and attempted to clarify its correlation with Vip3Aa virulence. We found PHB2 could bind directly to Vip3Aa to facilitate its internalization into Sf9 cells. As a multifunctional protein, PHB2 also contributes to maintaining mitochondrial stability. Downregulation of *phb2* expression in Sf9 cells rendered mitochondria more vulnerable and the cells more sensitive to Vip3Aa.

## Results

### PHB2 interacts with Vip3Aa

**Figure 1.**
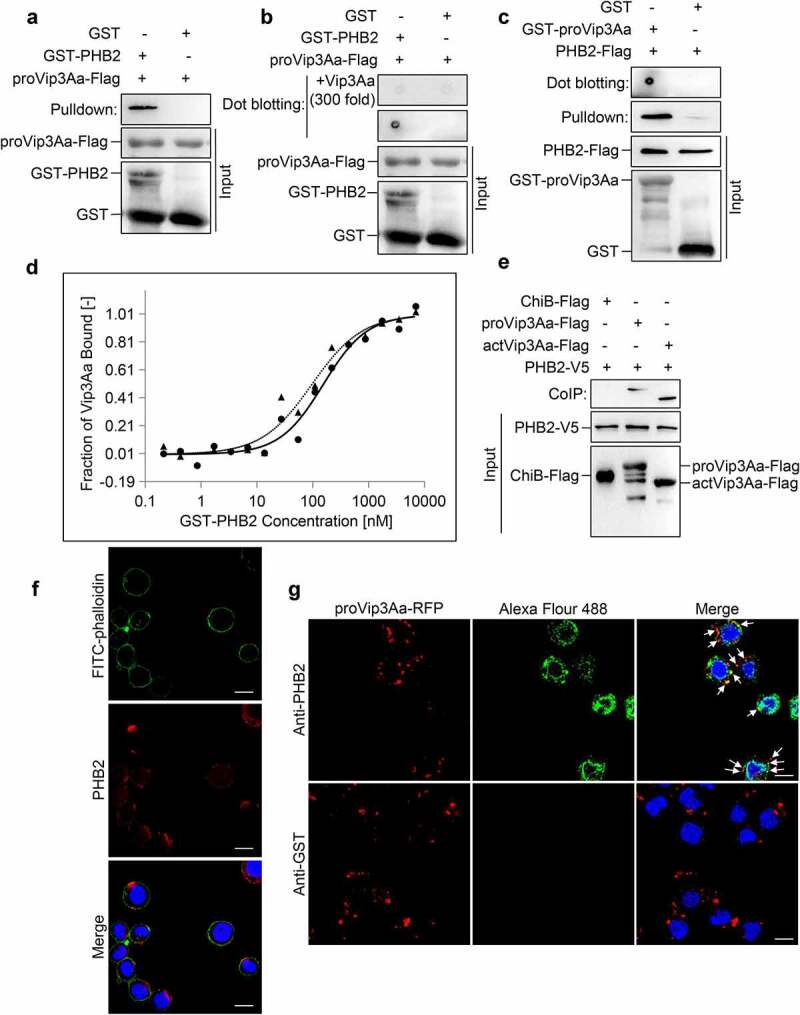
PHB2 interacts with Vip3Aa. (a) proVip3Aa-Flag was mixed with GST-PHB2 or GST and glutathione sepharose 4B beads successively. After washing 5 times the beads were used for immunoblotting and anti-Flag antibody was used to detect the proVip3Aa-Flag on beads. (b) GST-PHB2 and GST were dotted on a PVDF membrane and the membrane was incubated with Vip3Aa-Flag. In the competitive experiment, the membrane already dotted with GST-PHB2 or GST was incubated with proVip3Aa-Flag plus 300-fold proVip3Aa without Flag tag. Then the proVip3Aa-Flag bound on PVDF membrane was detected with anti-Flag antibody. (c) PHB2-Flag was incubated with GST-proVip3Aa or GST and glutathione sepharose 4B beads successively, then the beads were washed 5 times followed by immunoblotting. The PHB2-Flag bound to the beads was detected with anti-Flag antibody. For dot blotting, GST-proVip3Aa and GST were dotted on a PVDF membrane directly and the membrane were incubated with PHB2-Flag. The PHB2-Flag bound to the PVDF membrane was detected with anti-Flag antibody. (d) MST assay to measure the binding affinity between Vip3Aa and PHB2. the labeled proVip3Aa and actVip3Aa were kept constant at 333 nM and 216 nM respectively, and the GST-PHB2 was titrated from 0.2 nM to 7 μM. the solid line and circular sign mean proVip3Aa fit and dose response respectively and the dotted line and triangle sign mean actVip3Aa fit and dose response respectively. (e) Sf-PHB2 cell lysate was incubated with proVip3Aa-Flag, actVip3Aa-Flag, or ChiB-Flag, and then 5 μL of rabbit anti-V5 antibody and 40 μL of protein G agarose beads were added successively. The beads were washed 5 times and the Flag tagged protein bound to the beads was detected with anti-Flag antibody. (f) PHB2 (red) was stained with anti-PHB2 polyclonal antibodies to show its localization in Sf9 cells. DAPI (blue) and FITC-phalloidin (green) were used to stain the nuclei and cell membrane respectively. Scale bar, 20 μm. (g) The localization of proVip3aa-RFP (red) and PHB2 (green) in Sf9 cells was observed by a confocal microscopy. PHB2 was stained with anti-PHB2 polyclonal antibodies. In control group, anti-GST polyclonal antibodies were used. the co-localization between PHB2 and Vip3Aa was point at by arrows. Nuclei were stained using DAPI (blue). Scale bar, 20 μm.

To determine whether there is an interaction between PHB2 and Vip3Aa, we used purified glutathione-S-transferase (GST)-PHB2 and Vip3Aa-Flag protoxin (proVip3Aa-Flag) for pull-down and dot blotting analysis, with GST as a control. The results demonstrated proVip3Aa-Flag could bind to GST-PHB2 rather than GST (Figure 1a,b). The results of the competitive assay showed that excess proVip3Aa (300-fold, without Flag tag) could competitively bind to GST-PHB2 and affect the binding between proVip3Aa-Flag and GST-PHB2 (Figure 1b). We also purified GST-Vip3Aa protoxin (GST-proVip3Aa) and PHB2-Flag. Pull-down and dot blotting experiments demonstrated that PHB2-Flag could bind to GST-proVip3Aa, but not to GST (Figure 1c).

To measure the binding affinity between Vip3Aa and PHB2, the proVip3Aa and the activated toxin of Vip3Aa (actVip3Aa) were labeled and their dissociation constants (Kd) were detected in phosphate-buffered saline (PBS). The Kd value of proVip3Aa with PHB2 was 45.9 nM and that of actVip3Aa with PHB2 was 30.9 nM (Figure 1d). These results suggest proVip3Aa and actVip3Aa have a similar binding affinity to PHB2. The activation of Vip3Aa did not affect the binding affinity.

The interaction between PHB2 and Vip3Aa was also probed using co-immunoprecipitation (CoIP) experiments. A stable Sf-PHB2 cell line expressing PHB2 with a V5 tag at its C-terminus was established. The Sf-PHB2 cells were harvested and lysed before incubation with Vip3Aa-Flag protein. The results showed both proVip3Aa-Flag and actVip3Aa-Flag could be co-immunoprecipitated with PHB2-V5. In the control group, chitinase B (ChiB)-Flag failed to co-immunoprecipitate with PHB2-V5 (Figure 1e).

### Vip3Aa co-localizes with PHB2 in Sf9 cells

As shown in Figure S1, the specificity and effectiveness of the PHB2 antibody were verified. To investigate whether PHB2 is located on the Sf9 cell surface, Sf9 cells were incubated with the PHB2 antibody followed by Alexa Fluor 594-conjugated antibody to show its distribution. Sf9 cells were also treated using fluorescein isothiocyanate (FITC)-phalloidin to show the plasma membrane, as described by Jiang et al [20]. The results are represented in Figure 1(f). PHB2 was observed to be located both on the cell membrane and in the cytoplasm.

The results of colocalization analysis demonstrated that proVip3Aa-RFP (red dots) co-localized with PHB2 (green dots) in Sf9 cells, as shown in Figure 1(g). In contrast, almost no green fluorescent representing GST was detected in the control group.

### PHB2 interacts with receptor SR-C

**Figure 2.**
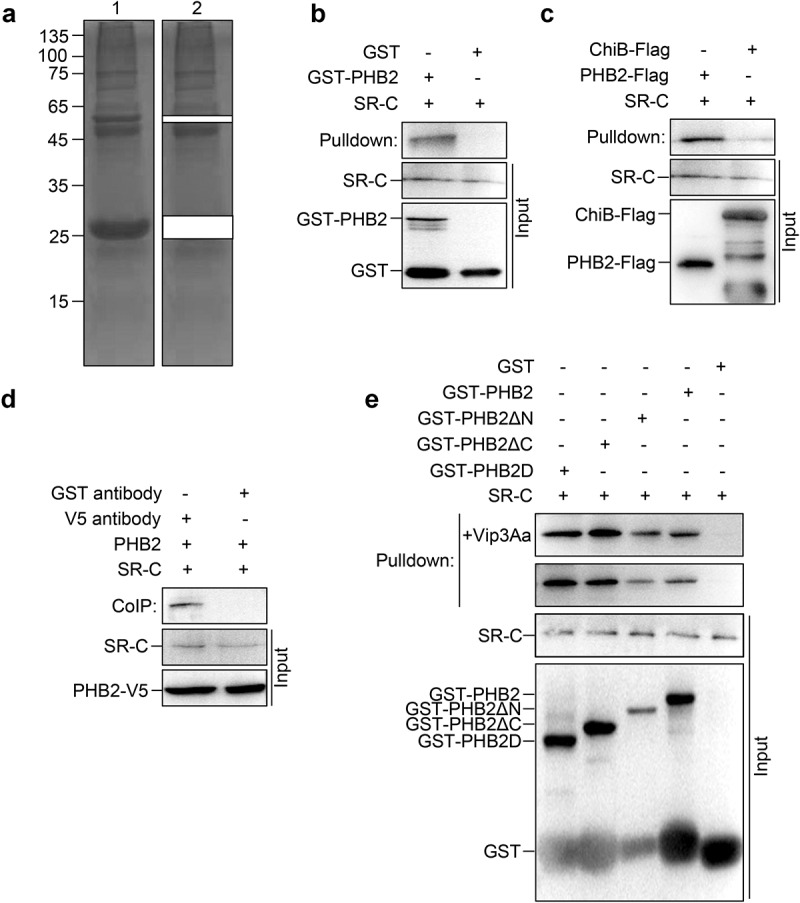
PHB2 interacts with receptor SR-C. (a) GST-PHB2 was incubated with Sf9 cell lysate and glutathione sepharose 4B beads successively. The beads were washed 5 times and detected by SDS-PAGE. Lane 1, the immune complexes. Lane 2, the remainder after GST and GST-PHB2 were excised. (b) The Sf9 cell lysate was incubated with GST-PHB2 or GST and glutathione sepharose 4B beads successively, then the beads were washed 5 times followed by an immunoblotting. The SR-C protein bound to the beads was detected with anti-SR-C antibody. (c) The Sf9 cell lysate was incubated with PHB2-Flag or ChiB-Flag, and then 5 μL of mouse anti-Flag antibody and 40 μL of protein G agarose beads was added successively. The beads were washed 5 times followed by an immunoblotting and the SR-C protein bound to the beads was detected with anti-SR-C antibody. (d) The Sf-PHB2 cell lysate was incubated with 5 μL of rabbit anti-V5 antibody and 40 μL of protein G agarose beads to immunoprecipitate SR-C. The beads were washed 5 times followed by an immunoblotting and the SR-C protein bound to the beads was detected with anti-SR-C antibody. (e) GST-PHB2, GST-PHB2ΔN, GST-PHB2ΔC, GST-PHB2D, and GST were incubated with Sf9 cell lysate and glutathione sepharose 4B beads successively respectively. The beads were washed 5 times followed by an immunoblotting and the SR-C protein bound to the beads was detected with anti-SR-C antibody.

To understand the role of PHB2 in the activity of Vip3Aa, we attempted to identify other proteins that interact with PHB2. Sf9 cell lysates were incubated with GST-PHB2, followed by immunoprecipitation with glutathione sepharose 4B beads. The protein complex was staining by Coomassie brilliant blue and the bands corresponding to GST and GST-PHB2 were excised followed by high-performance liquid chromatography-tandem mass spectrometry (HPLC-MS/MS) analysis (Figure 2a).

The MS/MS spectra revealed that the bands mainly contained mitochondrial proteins, nucleolus proteins, and cargo transport-associated proteins. We found SR-C was also screened as an interacting protein of PHB2 (S1 Dataset). Thus, the interaction between PHB2 and SR-C was detected using pull-down and CoIP analysis.

For the pull-down analysis, Sf9 cell lysates were incubated with GST-PHB2 or GST and glutathione sepharose 4B beads successively. SR-C was detected in the protein complex using SR-C polyclonal antibodies prepared previously [20]. These results demonstrated that there was an interaction between SR-C and GST-PHB2 but not between SR-C and GST (Figure 2b).

For the other pull-down analysis, Sf9 cell lysates were incubated with PHB2-Flag, anti-Flag antibody, and protein G agarose beads successively, and ChiB-Flag was used as control. The results showed in Sf9 cells that SR-C could be co-immunoprecipitated with PHB2-Flag, but not with ChiB-Flag (Figure 2c). In CoIP analysis, Sf-PHB2 cells lysates were incubated with anti-V5 antibodies and protein G agarose beads successively, and an anti-GST antibody was used in the control group. Results showed that PHB2-V5 could interact with SR-C (Figure 2d). Collectively, these results suggested that there was an interaction between PHB2 and SR-C *in vivo*.

### PHB2 interacts with SR-C though its SPFH domain

BLASTP analysis showed that PHB2 from *Spodoptera frugiperda* shares 72% amino acid sequence identity with human PHB (NP_001138303.1), and both contain an SPFH domain. To determine which fragments of PHB2 are involved in binding with SR-C, we constructed several mutant PHB2 proteins: GST-PHB2ΔN without the first 40 amino acids at the *N*-terminus (aa 41–299), GST-PHB2ΔC without 71 amino acids at the C-terminus (aa 1–228), and GST-PHB2D containing the core SPFH domain (aa 41–228) only.

Pull-down assays were conducted by incubating the mutant proteins with Sf9 cell lysates separately. The results showed that GST-PHB2, GST-PHB2ΔN, GST-PHB2ΔC, and GST-PHB2D could interact with SR-C, while GST could not (Figure 2e). This suggested that PHB2 interacts with SR-C through its SPFH domain. Moreover, the presence or absence of proVip3Aa did not affect the interaction between SR-C and PHB2. These results suggested that the interaction between SR-C and PHB2 was inherent rather than initiated by proVip3Aa (Figure 2e).

### Reducing PHB2 expression in Sf9 cells affects the internalization of Vip3Aa

**Figure 3.**
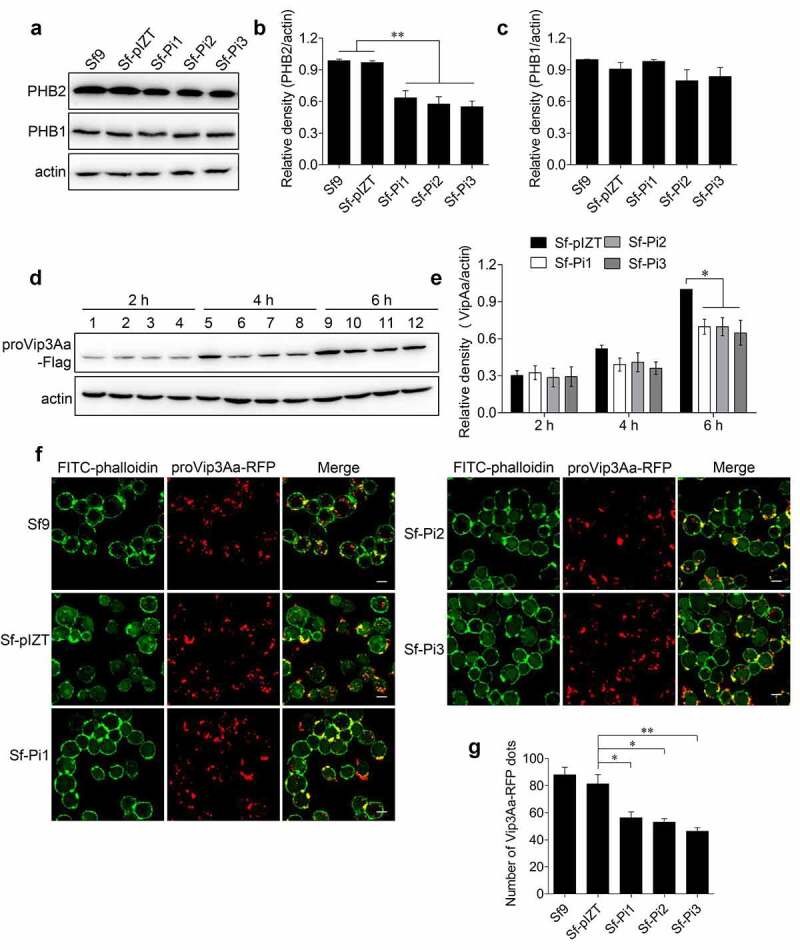
PHB2 affects Vip3Aa internalization. (a) PHB2, PHB1, and actin protein levels of Sf9, Sf-pIZT, Sf-Pi1, Sf-Pi2, and Sf-Pi3 cell lines were detected using immunoblotting analysis. (b) Relative densitometry analysis of PHB2/actin. (c) Relative densitometry analysis of PHB1/actin. (d) The content of proVip3Aa-Flag in Sf-pIZT, Sf-Pi1, Sf-Pi2, and Sf-Pi3 cells was detected using immunoblotting analysis. Each cell line was pre-treated withproVip3Aa-Flag (20 μg/mL) for 2, 4, or 6 h. Lane 1, 5, and 9: Sf-pIZT cell line. Lane 2, 6, and 10: Sf-Pi1 cell line. Lane 3, 7, and 11: Sf-Pi2 cell line. Lane 4, 8, and 12: Sf-Pi3 cell line. (e) Relative densitometry analysis of proVip3Aa/actin. (f) The proVip3Aa-RFP dots in each cell line were observed by a confocal microscopy. Each cell line was pre-treated with Vip3Aa-RFP (5 μg/mL) for 6 h and FITC-phalloidin (green) was used to stain cell membrane. Scale bar, 10 μm. (g) Using a blind fashion to quantitate the number of conspicuous Vip3Aa-RFP dots in each cell line (n = 50 cells per sample). Data are expressed as the mean ± SD from three independent experiments; ns, non-significant; * p < 0.05, ** p < 0.01, and ***p < 0.001.

Three plasmids to generate fragments of double-strand RNA (dsRNA) of the *phb2* gene, pIZT-PHB2i1, pIZT-PHB2i2 and pIZT-PHB2i3 were successfully transfected into Sf9 cells separately to knockdown the expression of *phb2* in Sf9 cells, and stable cell lines Sf-Pi1, Sf-Pi2 and Sf-Pi3 were established accordingly. The Sf-pIZT cell line was also constructed by transfected the Sf9 cells with the void plasmid pIZT/V5-HisB. The endogenous PHB2 protein levels in Sf-Pi1, Sf-Pi2, and Sf-Pi3 cells were reduced significantly (Figure 3a,b).

It was reported the contents of PHB1 and PHB2 proteins in Huh7.5.1 cells are interdependent [23]. Considering that knockdown of *phb2* expression level may affect the expression of *phb1*, in order to rule out the effect of reduced *phb1* expression on the internalization of Vip3Aa, we also measured the content of PHB1 in each cell line. The results of immunoblotting showed the level of PHB1 in each cell line was almost unchanged (Figure 3c).

To verify whether PHB2 has a role in Vip3Aa internalization, proVip3Aa-Flag (20 μg/mL) was added to the medium of Sf-pIZT, Sf-Pi1, Sf-Pi2, and Sf-Pi3 cells and incubated for 2, 4 and 6 h, separately. The internalized proVip3Aa-Flag in each cell line was detected using an anti-Flag antibody. The results showed at 2 h, the content of proVip3Aa-Flag in Sf-Pi1, Sf-Pi2, and Sf-Pi3 cells was similar to that in Sf-pIZT cells. At 4 and 6 h, the content of proVip3Aa-Flag in Sf-Pi1, Sf-Pi2, and Sf-Pi3 cells decreased compared with that in Sf-pIZT cells (Figure 3d,e).

To confirm this result, proVip3Aa-RFP (5 μg/mL) was added to Sf9, Sf-pIZT, Sf-Pi1, Sf-Pi2, and Sf-Pi3 cells medium and incubated for 6 h followed by an observation using confocal laser scanning microscopy (CLSM). The number of clear and distinct red dots of each cell line was counted. The results showed that the internalization of proVip3Aa-RFP in Sf-Pi1, Sf-Pi2, and Sf-Pi3 cells was decreased compared with that in Sf-pIZT and Sf9 cells (Figure 3f,g).

### Reducing PHB2 expression in Sf9 cells affects the virulence of Vip3Aa

**Figure 4.**
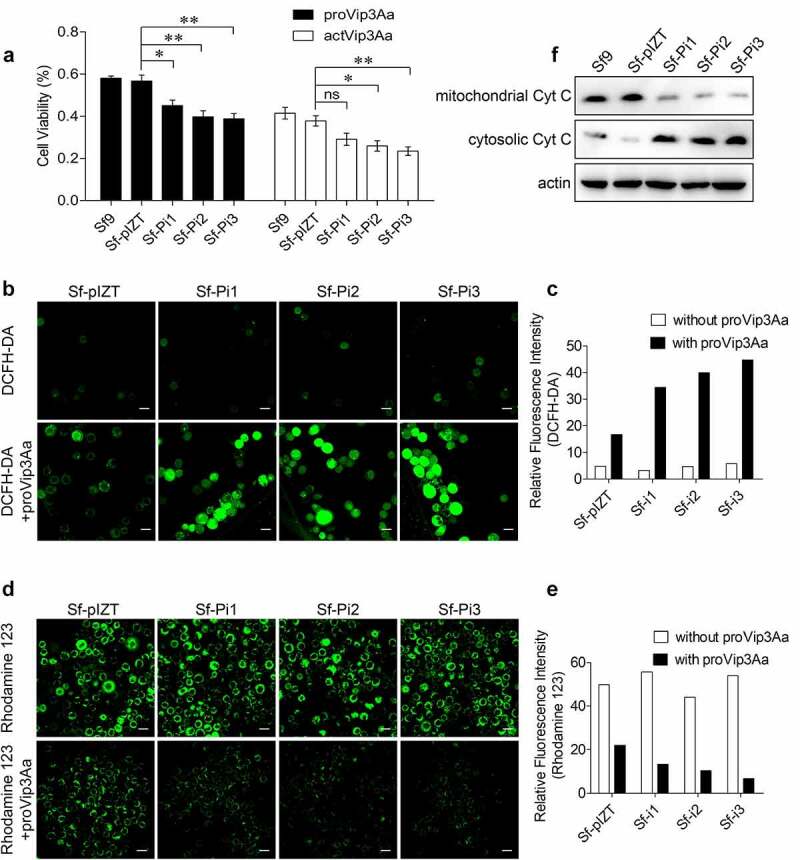
PHB2 affects mitochondrial function. (a) Cell viability of each cell line exposed to 50 μg/mL of proVip3Aa or 30 μg/mL of actVip3Aa for 48 h. Ns, non-significant; * p < 0.05, ** p < 0.01. (b) ROS levels of Sf-pIZT, Sf-Pi1, Sf-Pi2, and Sf-Pi3 cell lines exposed to 50 μg/mL of proVip3Aa for 24 h and DCFH-DA was used to detect the ROS level. Scale bar, 20 μm. (c) Relative fluorescence intensity of DCFH-DA in Sf-pIZT, Sf-Pi1, Sf-Pi2, and Sf-Pi3 cell lines. (d) Mitochondrial membrane potential of Sf-pIZT, Sf-Pi1, Sf-Pi2, and Sf-Pi3 cell lines exposed to 50 μg/mL of proVip3Aa for 24 h and Rhodamine 123 was used to detect the mitochondrial membrane potential. Scale bar, 20 μm. (e) Relative fluorescence intensity of Rhodamine 123 in Sf-pIZT, Sf-Pi1, Sf-Pi2, and Sf-Pi3 cell lines. (f) Immunoblotting analysis of the subcellular distribution of cytochrome c in Sf-pIZT, Sf-Pi1, Sf-Pi2, and Sf-Pi3 cell lines.

To determine whether PHB2 affects the cytotoxicity of Vip3Aa toward Sf9 cells, proVip3Aa (50 μg/mL) and actVip3Aa (30 μg/mL) were added into the medium of Sf9, Sf-pIZT, Sf-Pi1, Sf-Pi2, and Sf-Pi3 cells respectively for an incubation of 48 h. The cytotoxic effects of proVip3Aa and actVip3Aa on each cell line was detected by a CCK-8 cytotoxicity assay kit. The result showed the mortality in Sf-Pi1, Sf-Pi2, and Sf-Pi3 cells were increased significantly compared with that in Sf-pIZT and Sf9 cells (Figure 4a). Knocking down the expression of SR-C and FGFR receptors led to reduced levels of internalized Vip3Aa and increased the viability of Sf9 cells against Vip3Aa [20,21]. The downregulation of *phb2* expression reduced the level of internalized Vip3Aa; however, it also resulted in a decrease in cell viability. This suggested that downregulation of *phb2* expression also affected other steps related to Vip3Aa toxicity in Sf9 cells.

### Downregulation of PHB2 expression aggravates mitochondrial damage

The functions of PHB1 and PHB2 was reported that are closely related to mitochondria, and the loss of PHBs has been shown to impair mitochondrial morphology [24,25]. Thus, the effect of the downregulating *phb2* expression on mitochondrial function was investigated under conditions of Vip3Aa treatment.

After being treated with proVip3Aa for 24 h, the reactive oxygen species (ROS) level, mitochondrial membrane potential, and cytochrome c distribution in Sf9, Sf-pIZT, Sf-Pi1, Sf-Pi2, and Sf-Pi3 cells were determined [26,27]. Two fluorescent probes, dichloro-dihydro-fluorescein diacetate (DCFH-DA) and Rhodamine 123, were used to detect intracellular ROS and mitochondrial membrane potential, respectively. Results showed Sf-Pi1, Sf-Pi2, and Sf-Pi3 cells have a significantly higher ROS levels than Sf-pIZT cells (Figure 4b,c) and the mitochondrial membrane potential of Sf-Pi1, Sf-Pi2, and Sf-Pi3 cells was significantly lower than that in Sf-pIZT cells after proVip3Aa treatment (Figure 4d,e).

The results of immunoblotting showed that the contents of cytochrome c in the cytoplasm of Sf-Pi1, Sf-Pi2, and Sf-Pi3 cells were markedly higher than those in Sf9 and Sf-pIZT cells after proVip3Aa treatment, while the situation in mitochondria was the opposite (Figure 4F). These results suggested that PHB2 in mitochondria contributes to maintaining mitochondrial function. Therefore, downregulation of *phb2* expression aggravated the mitochondrial damage induced by Vip3Aa in Sf9 cells.

## Discussion

Vip3A proteins are efficient alternatives to Cry toxins and have been used widely against common pests, such as *Agrotis ipsilon*, *Helicoverpa armigera*, *Spodoptera exigua*, *Spodoptera frugiperda*, *Heliothis virescens*, and *Manduca sexta* [2,10,28–31]. More importantly, because their insecticidal mechanism is different from Cry toxins, Vip3A proteins can be used with Cry toxins simultaneously to kill the same pest. This “pyramid” strategy can delay the evolution of insect resistance to Bt crops or Bt-bioinsecticide reagents [20,32]. Exploring the mechanisms of Vip3 toxins could enable us to use them rationally and efficiently.

The main steps of the Vip3A toxicity process in susceptible insects include proteolytic activation of Vip3A to enable the pore-formation ability and the specific binding to receptors on midgut epithelium cells. Binding to the receptors is necessary, though it may not be sufficient, for Vip3A to exert its potency [10,28,33–36]. SR-C and FGFR have been identified as receptors for Vip3Aa in Sf9 cells. However, whether there are other receptors is unclear. Identifying new receptors will help us better understand the mechanism of the action of Vip3A toxins to sensitive pests. In this work, we revealed that PHB2 functions as a receptor for Vip3Aa, which affects the internalization of Vip3Aa. In addition, PHB2 is involved in mitochondrial stability, affecting the virulence of Vip3Aa to Sf9 cells.

PHB proteins can inhibit cell proliferation, hence their name prohibitin [37]. PHB1 and PHB2 reside ubiquitously in cell compartments, including the nucleus, mitochondria, and cytosol, and perform different functions [38]. PHB1 and PHB2, as intracellular proteins, are associated with the antigen receptor IgM of mouse B lymphocytes and might contribute to the internalization of IgM-antigen receptor complexes [39]. However, in other cases, PHB1 and PHB2 were reported to be located at the cell surface and participate in the internalization of chronic hepatitis C virus [23], Vi capsular polysaccharide [40], dengue virus serotype 2 [41], and Enterovirus [42].

In this study, the distribution of PHB2 in Sf9 cells was detected. The results showed the PHB2 red dots could be observed in the cytoplasm and on the cell membrane. Moreover, the direct binding of PHB2 and Vip3Aa was verified using pull-down and dot blotting assays. Furthermore, co-localization of PHB2 and Vip3Aa was observed. These results suggested that PHB2 interacts with Vip3Aa as a receptor on the cell membrane. This is different from the chaperone S2 protein of Vip3A. S2 in Sf21 cells can interact with Vip3A in pull-down assays and co-localized with Vip3A. It was speculated that the interaction between S2 and Vip3A is more likely to occur in the cytoplasm because S2 should be a cytoplasmic protein. Therefore, although downregulating the expression of S2 improved the virulence of Vip3A to S21 cells, S2 is still referred to as the chaperone of Vip3A, not the receptor [18].

In addition, we found that SR-C could interact with PHB2 using HPLC-MS/MS method with PHB2 as the bait. This result was further confirmed by pull-down and CoIP analysis between PHB2 and SR-C. We speculated that PHB2 acts as a receptor of Vip3Aa to enrich the concentration of Vip3Aa on the cell surface. Once captured by PHB2, Vip3Aa might be delivered to SR-C, thereby facilitating its internalization. Further experimental evidence is needed to confirm this hypothesis.

Using 2-dimensional gel electrophoresis coupled with ligand blotting, Bayyareddy identified several Cry4Ba toxin-binding proteins (including PHB) from the brush border membrane of midgut of *Aedes aegypti*; however, the binding between PHB and Cry4Ba was not further confirmed using other assays [43]. In 2013, it was reported that the Cry3Aa toxin could bind to PHB1 in the *Leptinotarsa decemlineata* larval midgut membrane, and the combination of *phb1* RNAi and Cry toxins was an effective strategy to improve crop protection [44]. In 2021, it was reported PHB2 could interact with domain III of Cry1Ab and participate in the toxicity of *Helicoverpa armigera* [45]. Here, we demonstrated that PHB2 on the Sf9 cell surface could interact directly with Vip3Aa and promote its internalization. Whether PHB2 can bind to other toxins and how it promotes the virulence process need further research.

It has been reported that the endocytosis of Vip3Aa is related to the insecticidal activity. The more Vip3Aa endocytosis, the higher the cell mortality [20,21]. The content of Vip3Aa in Sf9 cells was reduced and the survival rate of Sf9 cells increased significantly when the receptors SR-C or FGFR were depleted. To our surprise, although the downregulation of PHB2 reduced the internalization of Vip3Aa into Sf9 cells, the mortality of Sf9 cells increased. Thus, we presumed that PHB2 is also involved in other cellular processes that affect cell mortality caused by Vip3Aa.

Hou et al. reported that Vip3Aa induced mitochondrial dysfunction, such as the collapse of the mitochondrial membrane potential, the accumulation of ROS and the release of cytochrome c [15]. In cells the abnormal accumulation of ROS would attack mitochondria, increase mitochondrial membrane permeability, and finally induce apoptosis of Sf9 cells [46,47]. Mitochondrial membrane potential and cytochrome c distribution were used to assess the status of mitochondria as indexes of early activation of apoptosis [48]. One of the important functions of PHB2 is to form heteroligomers with PHB1 in the inner membrane of mitochondria to maintain their stability [49]. We hypothesized that the downregulation of PHB2 expression might affect mitochondrial stability and aggravate mitochondrial damage after Vip3Aa treatment.

To test this hypothesis, we detected the changes in ROS levels, mitochondrial membrane potential, and cytochrome c distribution in Sf9 cells with down-regulated PHB2 levels after Vip3Aa treatment. When *phb2* expression was knocked down, the mitochondrial membrane potential decreased significantly. Meanwhile, the ROS levels were significantly higher, and more cytochrome c was released in Vip3Aa-treated Sf9 cells. Our observation was consistent with the report that over-expression of PHB could prevent decreases in mitochondrial membrane potential, ROS production, and leaking of cytochrome c from mitochondria to protect cells from MPP±induced neuronal death [50]. These results suggested that PHB2 could maintain mitochondrial stability, thus the downregulation of PHB2 aggravates mitochondrial damage induced by Vip3Aa, making Sf9 cells more sensitive to challenge by Vip3Aa.

PHB1 and PHB2 are highly conserved proteins that act interdependently [51]. It was reported the expression levels of both PHB1 and PHB2 were reduced in *phb1* RNAi treated *Aedes aegypti* CCL-125 cells [41]. Similarly, *phb1* and *phb2* knockdown decreased the protein levels of each other in Huh7.5.1 cells [23]. However, our results showed that the PHB1 level was almost unchanged in *phb2* knockdown cell lines, suggesting that the expression of PHB1 in Sf9 cells was not affected by the downregulation of PHB2. This result is consistent with Su et al. observation that the PHB1 expression was not changed significantly after *phb2* knockdown in human rhabdomyosarcoma cells [52]. We speculated that PHB1 expression is independent of PHB2, while a decrease in PHB2 expression is sufficient to disrupt the function of the PHB complexes in Sf9 cells.

As an important organelle in cells, mitochondria have essential functions in energy metabolism, ion homeostasis, the transmission of apoptosis signals, and so on. Mitochondria are related to many diseases like neurological disorders [53], cancer [54], Alzheimer’s disease [55] and cardiac ischemia-reperfusion injury [56–59]. We found that mitochondria also play a vital role in the pathway of Vip3Aa toxicity in insect cells. Downregulation of PHB2 in Sf9 cells caused two effects: decreased Vip3Aa internalization and more severe mitochondrial dysfunction. The cytotoxicity assay results suggested that the function of PHB2 in mitochondrial homeostasis is more important than its function in Vip3Aa internalization for Sf9 mortality. When mitochondrial function is impaired, limited amounts of Vip3Aa can cause higher cell mortality. Consequently, combining reagents that inhibit mitochondrial functions or RNAi targeting essential mitochondrial proteins with Vip3Aa will become an attractive strategy to improve the insecticidal effect with reduced resistance development of pests.

It should be pointed out that Sf9 cells come from the ovary of *S. frugiperda*, although they are helpful to study the action mechanism of Vip3Aa. Whether the results obtained from Sf9 cells can represent the *in vivo* situation of midgut epithelial cells needs to be verified. Jiang et al. confirmed the receptor function of SR-C in *S. exigua* and SR-C transgenic Drosophila larvae *in vivo*, like its function in Sf9 cells [20]. Our findings in this work require further verification in insects like *S. frugiperda* to test whether PHB2 can still perform the same function in midgut cells.

## Conclusions

We demonstrated two functions of PHB2 in Sf9 cells associated with Vip3Aa toxicity. Firstly, PHB2 on the cell membrane binds to Vip3Aa to promote its entry into the cell. Secondly, PHB2 maintains mitochondrial stability to resist the negative influences of Vip3Aa. The function of PHB2 in mitochondria is essential for the virulence of Vip3Aa. The results of this study will improve our understanding of the insecticidal mechanism of Vip3A and develop more effective pest control strategies in the future.

## Materials and methods

### Bacterial strains and insect cells

*Escherichia coli* strains DH5α were used for plasmid constructions and BL21 (DE3) were used for protein purification. Sf9 cells were cultured in Sf-900II medium (Gibco, Grand Island, NY, USA) at 27 ℃ containing 10% fetal bovine serum (FBS, Gibco).

### Chemicals

Radioimmunoprecipitation assay (RIPA) buffer (#9806S), rabbit anti-V5 (# 13202), rabbit anti-PHB1 (#2426T), mouse anti-Flag (#8146), rabbit anti-cytochrome c (#11940T), and Alexa Fluor 488-conjugated goat anti-rabbit IgG (#4412) antibodies were purchased from Cell Signaling Technology (Danvers, MA, USA). Rabbit anti-GST (#AE067), rabbit anti-actin (#AC004), rabbit anti-PHB2 (#4504), Alexa Fluor 594-conjugated goat anti-rabbit IgG (#AS039), Alexa Fluor 594-conjugated goat anti-mouse IgG (#AS054), horseradish peroxidase (HRP)-conjugated goat anti-mouse IgG (#AS003), HRP-conjugated goat anti-rabbit IgG (#AS014) antibodies, and FITC-phalloidin (#RM02836) were obtained from ABclonal (Wuhan, China). Rabbit polyclonal anti-GST (#bs-3725 R) antibodies were obtained from Santa Cruz Biotechnology (Santa Cruz, CA, USA). 4′,6-diamidino-2-phenylindole (DAPI) was purchased from Sigma (St. Louis, MO, USA). Cellfectin® II Reagent and PLUS^TM^ Reagent were obtained from Invitrogen (Carlsbad, CA, USA). Rhodamine 123 (#C2007) and DCFH-DA (#S0033) were obtained from Beyotime Biotechnology (Nanjing, China). Prestained protein marker (#M221) was purchased from Genstar (Beijing, China).

### Protein purification

The *phb2* gene (GenBank accession no. HQ337624.1) from Sf9 cells was amplified using primers pETPHB2F and pETPHB2R to insert into the pET-28a(+) vector and was amplified using primers pGEXPHB2F and pGEXPHB2R to insert into pGEX-4T-1 vector using pEASY®-Uni Seamless Cloning and Assembly Kit (TransGen, Beijing, China) resulting in Flag-His_6_ and GST fusion proteins, respectively. Primers pGEXPHB2F, pGEXPHB2R, pGEXPHB2DF and pGEXPHB2DR were used to amplify *phb2* gene fragments to express truncated GST-PHB2 proteins. *E.coli* BL21 (DE3) was transformed with the recombined plasmids to express proteins. The BL21(DE3) strains could express Vip3Aa, Vip3Aa-RFP, Vip3Aa-Flag, ChiB-Flag was already built [20]. His_6_-tagged proteins were purified using an Ni Sepharose affinity column and GST-tagged proteins were purified using a GST-Sepharose affinity column (GE Healthcare, Fairfield, CT, USA). The dialysis step of purified proteins was performed as described previously [20].

All the primers used in this study are listed in Table 1.

**Table 1.** Primers used in this study

Primer Name	Sequence (5’-3’)
pETPHB2F	ACTTTAAGAAGGAGATATACATGGCACAAAGTAAGCTTAACGATATG
pETPHB2R	GGTGGTGCTCGAGCTTATCGTCGTCATCCTTGTAATCCTTCTTCTTAGTCAGCTTCTCG
pGEXPHB2F	GTTCCGCGTGGATCCCCGGAAATGGCACAAAGTAAGCTTAACGATAT
pGEXPHB2R	GTCACGATGCGGCCGCTCGACTTCTTCTTAGTCAGCTTCTCGGAC
pGEXPHB2DF	GTGGATCCCCGGAATCCTTGTTTACCGTT
pGEXPHB2DR	CAGTCACGATGCGGCCGCTCGAGCTACTGTACAATCTTTTGTTGAC
pGEXVipF	GGGCCCCTGGGATCCCCGGAAATGAACAAGAATAATACTAAATTAAGC
pGEXVipR	CAGTCACGATGCGGCCGCTCCTACTTAATAGAGACATCGTAAAAATG
pIZTPHB2F	TCGAATTTAAAGCTTGGTACATGGCACAAAGTAAGCTTAAC
pIZTPHB2R	CTCTAGACTCGAGCGGCCCTTCTTCTTAGTCAGCTTCTCGGAC
Pi1UPF	TCGAATTTAAAGCTTGGTACATGGCACAAAGTAAGCTTAACGATA
Pi1UPR	GTTGCAAAGTTCAATAGCTTCAACTGCAGCTGTGTATTCC
Pi1DOF	GCTGCAGTTGAAGCTATTGAACTTTGCAACCACAGACTTC
Pi1DOR	GTGATGGTGATGATGACCGGATGGCACAAAGTAAGCTTAACGATA
Pi2UPF	TCGAATTTAAAGCTTGGTACGACTATGATGAAAAAGTGTTGCCCT
Pi2UPR	GGGTAAAGCTATGGGAGGAATACACGGTTTTGAGATTGGG
Pi2DOF	AAACCGTGTATTCCTCCCATAGCTTTACCCAACATTTCAG
Pi2DOR	GTGATGGTGATGATGACCGGGACTATGATGAAAAAGTGTTGCCCT
Pi3UPF	TCGAATTTAAAGCTTGGTACGCAACACGTTATGAGTGAAGGTATG
Pi3UPR	TGGTAAGGAATACACCTGTACAATCTTTTGTTGACGCTCC
Pi3DOF	CAAAAGATTGTACAGGTGTATTCCTTACCAAAGCTCAATT
Pi3DOR	GTGATGGTGATGATGACCGGGCAACACGTTATGAGTGAAGGTATG

### Vip3Aa protein activation

The purified 89 kDa full-length proVip3Aa was incubated with trypsin at a 1:100 ratio (trypsin: Vip3Aa, *w:w*) in PBS buffer at 28 ℃ for 2 h. One milliliter of protein mixture was then concentrated to 100 μL using a Amicon Ultra-15 Centrifugal Filter (100 kDa molecular weight cutoff) Devices (EMD Millipore, Billerica, MA, USA). Then, 900 μL of PBS was added to reach the volume of 1 mL. The concentration steps were repeated three times to remove trypsin.

### Microscale Thermophoresis (MST) assay

The proVip3Aa or actVip3Aa dialyzed against PBS (pH 7.5) were labeled using a Monolith NT^TM^ Protein Labeling Kit (#L008, NanoTemper Technologies GmbH, Munich, Germany). Labeled proVip3Aa (333 nM) or labeled actVip3Aa (216 nM) was incubated with 0.2 nM to 7 μM GST-PHB2 protein. Then the protein complex was loaded into capillaries and analyzed using a NanoTemper® Monolith NT.115 Pico (NanoTemper Technologies GmbH). The MO Affinity Analysis v2.2.2 software (NanoTemper Technologies GmbH) was used to normalize the fluorescence signal and fit the Kd equation.

### Plasmid construction and transfection

The *phb2* gene was amplified using primers pIZTPHB2F and pIZTPHB2R and inserted into pIZT/V5-HisB vector to express a PHB2-V5 fusion protein in Sf9 cells. To silence the *phb2* gene in Sf9 cells, fragments of the *phb2* gene (nucleotides [nt] 1–600, dsRNA1s) were amplified using primers Pi1UPF and Pi1UPR and the complementary strand (nt 450–1, dsRNA1as) was amplified using primers Pi1DOF and Pi1DOR. The pIZT-Pi1 vector was constructed by inserting dsRNA1s and dsRNA1as into the pIZT/V5-HisB vector in tandem which was digested by *Kpn*I-*Age*I. The plasmids pIZT-Pi2 or pIZT-Pi3 were constructed similarly to pIZT-Pi1 using primers Pi2UPF/R and Pi2DOF/R or Pi3UPF/R and Pi3DOF/R. The pIZT/V5-HisB, pIZT-Pi1, pIZT-Pi2 or pIZT-Pi3 plasmids was transfected into Sf9 cells to generate Sf-pIZT, Sf-Pi1, Sf-Pi2, and Sf-Pi3 cell lines. About a four-weeks selection of Zeocin (300 μg/mL), the expression level of the endogenous PHB2 protein was detected using anti-PHB2 antibodies and the PHB2-V5 expression level was detected using anti-V5 antibodies.

### Dot blotting, pull-down, and co-immunoprecipitation

For dot blotting, 5 μL of GST-PHB2 and GST (100 μg/mL) were separately dotted onto a PVDF membrane. Then 5% skimmed milk was used to block the membrane for 1 h followed by an incubation with 5% skimmed milk containing Vip3Aa-Flag (100 μg/mL) for 1.5 h at room temperature. In the competition binding experiments, a 200-fold excess Vip3Aa without a Flag tag was used for incubation. Then, the PVDF membrane was incubated with anti-Flag antibodies and anti-mouse second antibodies, successively.

For the pull-down assay, Vip3Aa-Flag (100 μg/mL) was incubated with GST-PHB2 (10 μg/mL) for 2 h and GST-Sepharose affinity beads for 2 h successively. Then the beads were washed for five times using PBS (with 0.5% Triton X-100). Western blotting method was used to detect the precipitated components.

The pull-down assay also used to detect the interaction between SR-C and PHB2. The Sf9 cells lysate in RIPA buffer was incubated with GST-PHB2 and glutathione sepharose 4B beads, successively. After washing five times in PBS, western blotting method was used to detect the precipitated components.

For co-immunoprecipitation, an incubation between Vip3Aa-Flag (100 μg/mL) and Sf-PHB2 cell lysate at 4 ℃ for 2 h. Then 5 μL of rabbit anti-V5 and 40 μL of protein G agarose beads (Santa Cruz) was added successively for an incubation of 2 h to immunoprecipitate proteins. Subsequently protein G agarose beads were washed five times with PBS (with 0.5% Triton X-100) and analyzed using western blotting. The procedure of co-immunoprecipitation of SR-C and PHB2-V5 was similar to the coimmunoprecipitation steps of PHB2-V5 and Vip3Aa-Flag.

### Immunostaining and confocal microscopy

Sf9 cells cultured in laser confocal microscopy culture dishes were treated with Vip3Aa-RFP (10 μg/mL) for 6 h and washed with PBS. Then the Sf9 cells were fixed with 4% paraformaldehyde at 37 ℃ for 30 min followed by permeabilized with 0.2% Triton X-100 at 37 ℃ for 30 min successively. The anti-PHB2 polyclonal antibodies and Alexa Fluor 488-conjugated antibodies were used successively for immunostain of the Sf9 cells. The FITC-phalloidin and DAPI were used to label the cellular cortical actin and nuclei respectively. To stain the PHB2 on the cell membrane surface, the permeabilization step was omitted. Cell images were captured using a Zeiss LSM710 confocal microscope (Carl Zeiss, Oberkochen, Germany).

### Mass spectrometry

GST-PHB2 (10 μg/mL) was incubated with Sf9 cell lysate for 2 h and 40 μL of GST-Sepharose affinity beads for 2 h successively. Then the beads were washed five times using washing buffer (PBS with 0.5% Triton X-100). The beads were resuspended with 10% SDS followed by an SDS-PAGE. The bands representing GST and GST-PHB2 was cut out and the rest were sent for LC-MS/MS analysis.

### Measurement of intracellular ROS and mitochondrial membrane potential

Twenty microliters of Vip3Aa (50 μg/mL) were added into the cell medium for an incubation of 24 h. After the incubation, the cell medium was replaced with fresh medium with DCFH-DA (10 mM) or Rhodamine 123 (50 nM) for a further 30 min incubation of these Sf9 cells. DCFH-DA is a cell permeable probe used to detect intracellular ROS while Rhodamine 123 is a permeable cationic yellow green fluorescent dye that can be used to detect mitochondrial membrane potential. The cells were washed with PBS followed by an observation by confocal microscopy. The fluorescence intensity of pictures of each cell line treated with DCFH-DA or Rhodamine 123 was measured using ImageJ software (v1.15k).

### Mitochondrial isolation of Sf9 cells

The mitochondria of Sf9 cells were isolated using mitochondrial isolation kit (Beyotime, Shanghai, China). The collected Sf9 cells were resuspended with 1 mL cold mitochondrial isolation buffer (containing 1 mM PSFM) and stood on ice for 15 min. Then, a 26-G needles was used for homogenization of the cell suspension for 14 times. After a centrifugation at 4 °C 600 × g for 10 min, the supernatant was transferred to a new tube and centrifuged at 4 °C 10,000 × g for 10 min. The mitochondria were present at centrifugation sediment. Then the supernatant was transferred into a new tube and centrifuged at 4 °C 12,000 × g for 10 min. The supernatant was used as the cytoplasmic lysate.

### Cytotoxicity assay

Cell viability was detected using a Cell Counting Kit-8 (CCK-8) assay (Dojindo, Kumamoto, Japan) according to the previously reported method by Jiang et al [20]. Briefly, 100 μL cell suspensions (1 × 10^5^ cells) was added into each well of 96-well plates. Ten microliters of proVip3Aa solution (500 μg/mL), actVip3Aa solution (300 μg/mL) or dialysis buffer was added to each well for an incubation of 48 h. Each well was added with 10 μL of CCK-8 reagent followed an incubation in the dark for 2 h. A microplate reader (PerkinElmer, Boston, MA, USA) was used to measure the absorbance of each well at 450 nm. Cell viability (%) = absorbance of the Vip3Aa treated group/the absorbance of the dialysis buffer treated group × 100%.

### Statistical analysis

All experiments were performed at least three times independently. All statistical data were calculated with GraphPad Prism (Version 5.01). Two-tailed test was used to compare the means of two groups. Significance of mean comparison is annotated as follow: ns, not significant; *P < 0.05; **P < 0.01; ***P < 0.001.

## Supplementary Material

Supplemental Material

## Data Availability

The authors confirm that the data supporting the findings of this study are available within the article and its supplementary materials.
