# Supplementary figures and images for: PHB2 affects the virulence of Vip3Aa to Sf9 cells through internalization and mitochondrial stability
Source: Virulence. 2022 Apr 20;13(1):684–97. doi: 10.1080/21505594.2022.2064596 (PMC9037526; doi:10.1080/21505594.2022.2064596)

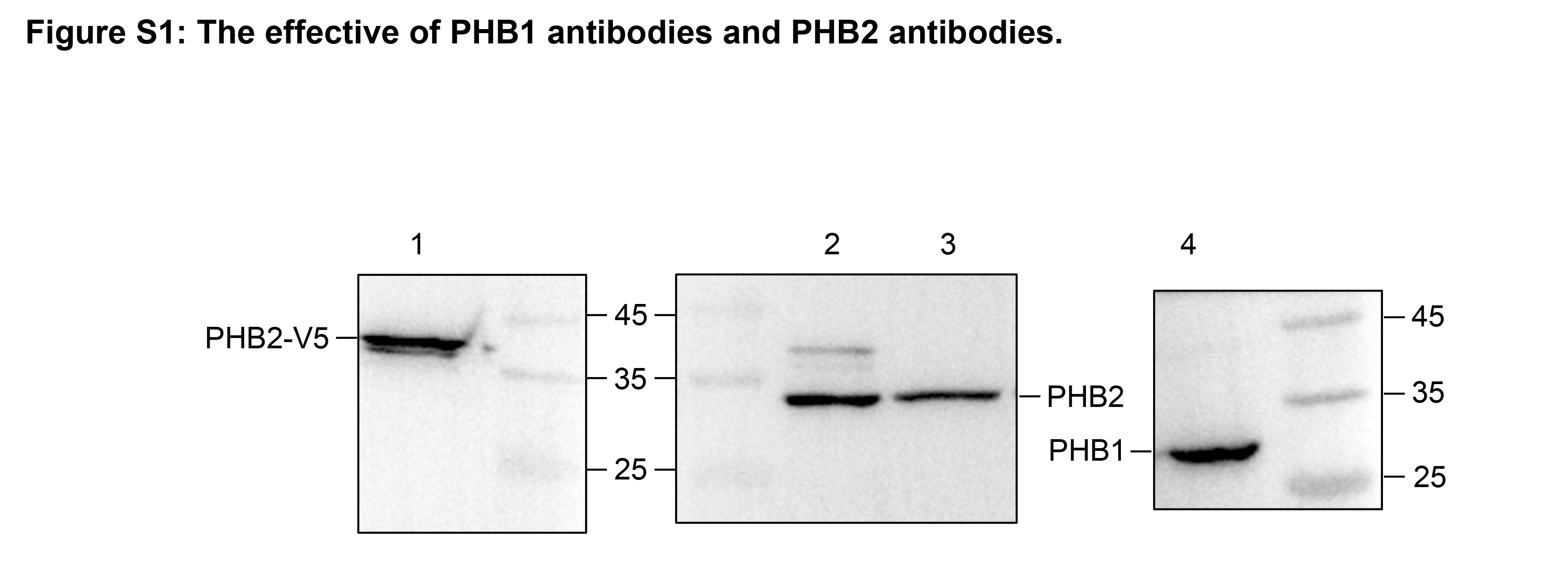

Supplement: Supplemental Material [file KVIR_A_2064596_SM3393.zip › supplementary/Figure S1 600.tif]

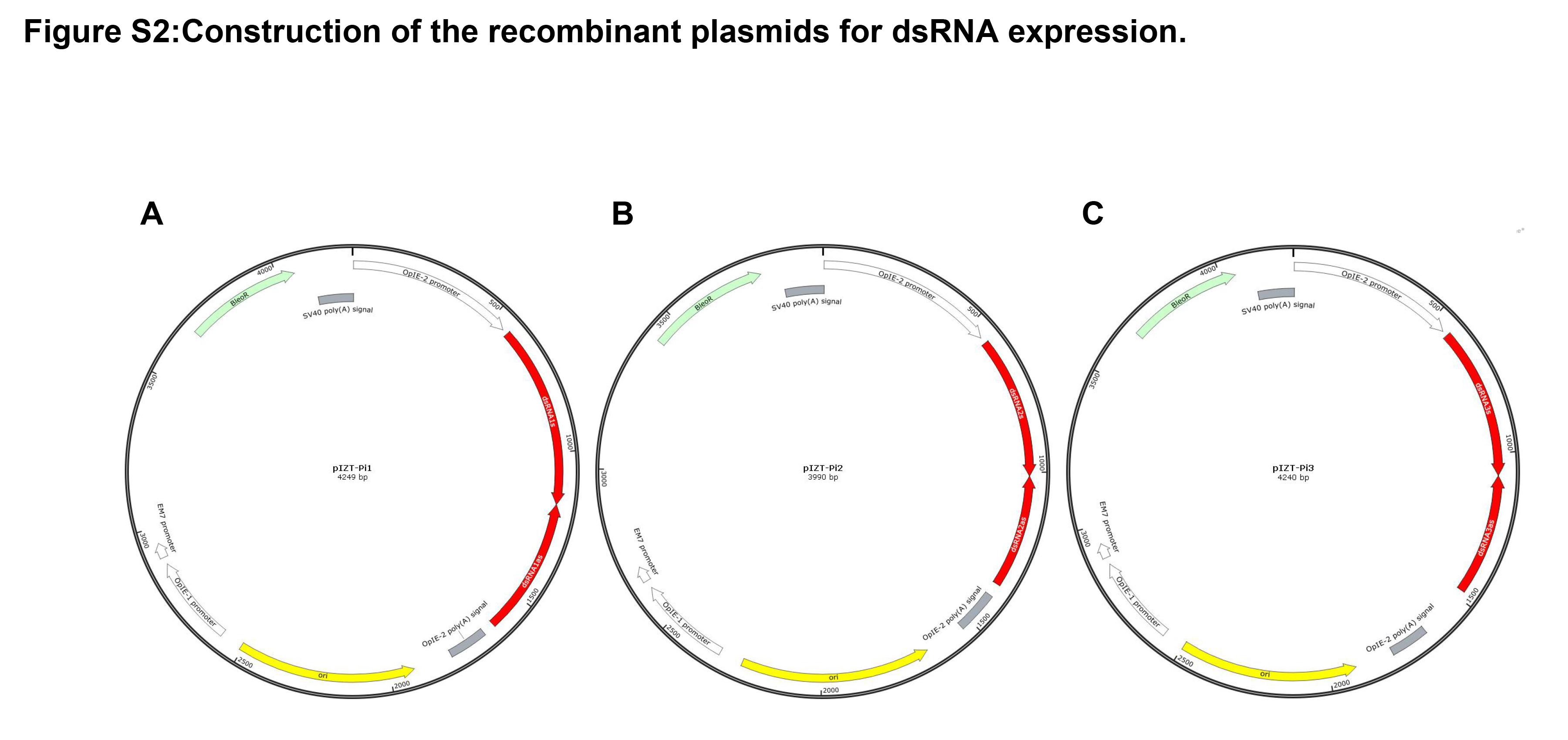

Supplement: Supplemental Material [file KVIR_A_2064596_SM3393.zip › supplementary/Figure S2 600.tif]
